# Glia and Orofacial Pain: Progress and Future Directions

**DOI:** 10.3390/ijms22105345

**Published:** 2021-05-19

**Authors:** Yi Ye, Elizabeth Salvo, Marcela Romero-Reyes, Simon Akerman, Emi Shimizu, Yoshifumi Kobayashi, Benoit Michot, Jennifer Gibbs

**Affiliations:** 1 Bluestone Center for Clinical Research, New York University College of Dentistry, New York, NY 10010, USA; Elizabeth.Salvo@nyulangone.org; 2 Department of Oral & Maxillofacial Surgery, New York University College of Dentistry, New York, NY 10010, USA; 3 Department of Molecular Pathobiology, New York University College of Dentistry, New York, NY 10010, USA; 4 Department of Neural and Pain Sciences, University of Maryland School of Dentistry, Baltimore, MD 21201, USA; mromero@umaryland.edu (M.R.-R.); sakerman@umaryland.edu (S.A.); 5 Department of Oral Biology, Rutgers School of Dental Medicine, Newark, NJ 07103, USA; shimize1@sdm.rutgers.edu (E.S.); kobayayo@rwjms.rutgers.edu (Y.K.); 6 Division of Endodontics in Restorative Dentistry and Biomaterials Sciences, Harvard School of Dental Medicine, Boston, MA 02115, USA; benoit_michot@hsdm.harvard.edu (B.M.); Jennifer_Gibbs@hsdm.harvard.edu (J.G.)

**Keywords:** Schwann cell, satellite ganglia cells, microglia, astrocytes, trigeminal ganglia, headache, toothache, dental pulp injury, head and neck cancer

## Abstract

Orofacial pain is a universal predicament, afflicting millions of individuals worldwide. Research on the molecular mechanisms of orofacial pain has predominately focused on the role of neurons underlying nociception. However, aside from neural mechanisms, non-neuronal cells, such as Schwann cells and satellite ganglion cells in the peripheral nervous system, and microglia and astrocytes in the central nervous system, are important players in both peripheral and central processing of pain in the orofacial region. This review highlights recent molecular and cellular findings of the glia involvement and glia–neuron interactions in four common orofacial pain conditions such as headache, dental pulp injury, temporomandibular joint dysfunction/inflammation, and head and neck cancer. We will discuss the remaining questions and future directions on glial involvement in these four orofacial pain conditions.

## 1. Introduction

**Figure 1:**
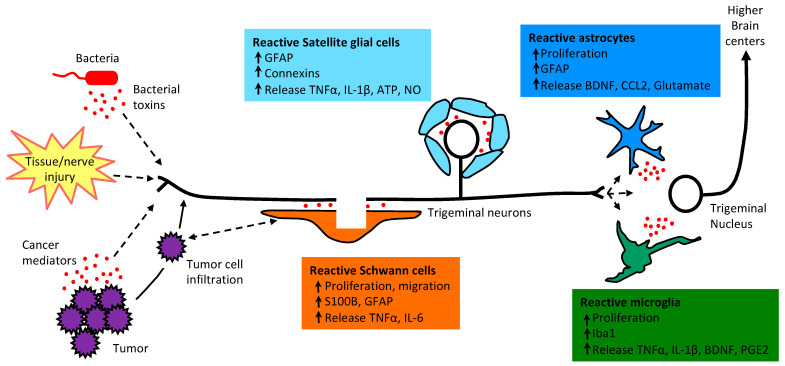
Functional alterations of glial cells, including Schwann cells, SGCs, astrocytes and microglia in response to rofacial tumors, bacterial toxins, dental pulp injury or tissue/nerve injury. Peripheral injury/inflammation induces an increase in glial cell proliferation and hypertrophy, changes in glial activation markers, and the release of pronociceptive mediators that can contribute to neuronal sensitization and pain. TNFα: tumor necrosis factor alpha, IL: interlukin; NO: nitrogen oxide; BDNF: brain derived neurotrophic factor; CCL2: C-C motif chemokine ligand 2; PGE2: prostaglandin E2.

Chronic orofacial pain is a major healthcare issue, affecting approximately 22% of Americans in adulthood and is associated with high morbidity and health service utilization [1,2]. Chronic orofacial pain conditions represent a challenge to clinicians because of the disease complexity and unclear etiology. Pain in the soft and hard tissues of the head, neck, and face is generally regarded as orofacial pain [2,3]. Orofacial structures include many unique end organs such as teeth, facial skin, intraoral mucosa, intracranial meninges, and temporomandibular joint (TMJ) with highly specialized anatomy and innervation [2,3,4]. Nerves and blood vessels heavily supply the orofacial region. Orofacial pain can be musculoskeletal, inflammatory, neuropathic, and vascular [3,4,5]. Cellular and molecular insights of orofacial pain are largely derived from animal models, yet behavioral phenotyping in the orofacial region is more challenging than other body regions (e.g., hind limb). The neuronal involvement of orofacial pain includes peripheral sensitization of the primary afferent neurons, central sensitization in the spinal cord and brain neurons, as well as the loss of inhibitory synaptic transmission and enhanced descending facilitation in the pain pathway, transitioning acute pain to chronic pain [6,7,8,9]. The non-neuronal mechanisms in pain, such as the contribution of glial cells, are not as well studied in the orofacial region compared to the other body regions [6]. Glial cells in the central nervous system (CNS) consist of three major types: astrocytes, microglia, and oligodendrocytes [4]. Glial cells in the peripheral nervous system (PNS) consist of satellite glial cells (SGCs) in the dorsal root ganglia (DRGs) and trigeminal ganglia (TGs), and Schwann cells in the peripheral nerve [4]. In response to tissue/nerve injury, bacterial infection, or tumor infiltration glial cells become reactivated and release mediators that could contribute to both peripheral and central sensitization (Figure 1). The most commonly used glial markers include calcium-binding protein beta (S100B) and glial fibrillary acidic protein (GFAP) that are expressed in Schwann cells, SGCs, and astrocytes, and ionized calcium-binding adaptor molecule 1 (Iba1), which is a microglia/macrophage-specific calcium-binding protein. CD11b or OX42 is often used to mark microglia as well.

Headaches, toothaches, TMJ dysfunction (TMD)/inflammation, and head neck cancer (HNC) pain are a few common orofacial pain conditions that can drastically impact the patient’s quality of life [5]. This review will provide an update on recent advances on glial cell involvement in these four orofacial pain conditions. Although dental pulpal injury and HNC are known to have a neuropathic component [10,11], existing studies do not distinguish glial activation by neuropathic vs. non-neuropathic mechanisms. For a focused review on glial mechanisms in orofacial neuropathic pain, please see a recent publication by Kuchukulla and Boison [12]. Model-specific glial responses induced by each of these pain conditions reviewed here are summarized in Table 1.

## 2. Headache

Primary headaches, such as migraines, can be chronic and disabling, affecting over a billion adults globally, with a huge socioeconomic burden [53]. Migraine is a neurovascular disorder where neural events happen in different peripheral structures, central areas of the brain, and brainstem [54,55,56]. The dura mater, meningeal blood vessels and their innervation provided by the ophthalmic branch of the trigeminal nerve, the afferent connection to the trigeminal nucleus caudalis (TNC), and a reflex connection from the TNC to the parasympathetic outflow to the cranial vasculature, including the dural meninges, are known as the trigeminovascular system [57,58]. The nociceptive afferent inputs from these structures transmit information to the TNC, brainstem, and higher processing centers [58]. Migraine and other primary headache disorders are present as a multi-symptom complex, where pain is not the only symptom. The symptomatology may be present with sensory symptoms (e.g., photophobia, phonophobia, cutaneous allodynia), autonomic symptoms (e.g., nausea, vomiting, rhinorrhea, lacrimation, ptosis), affective symptoms (e.g., irritability), and cognitive symptoms (e.g., transient amnesia, aphasia) [54]. In 30% of patients, migraine headache is also associated with fully reversible neurological symptoms called ‘migraine aura’ [59], which affect visual, sensory, speech and/or language, motor, brainstem or retinal systems. The symptoms of primary headaches are also influenced by environmental factors such as stress, fasting, hormonal fluctuations and sleep, suggesting the involvement of epigenetic mechanisms in primary headaches [60].

Glial involvement in headache disorders is evidenced by the ability of glial cells to modulate neuronal function by releasing mediators and phenotypic changes in response to headache [61]. Microglia release of inflammatory mediators, as well as astrocytic modulation of glutamatergic mechanisms, have been shown to likely influence the activation of multiple brain and brainstem regions relevant to migraine and other primary headache disorders [61,62,63,64,65]. The neuron–glia communication via gap junctions within the TG from in vitro studies provides evidence that neuronal calcitonin gene-related peptide (CGRP), a key neuropeptide that is released during migraine attacks and a target for newly approved migraine therapeutics, could cause the activation of the adjacent SGCs to release cytokines and chemokines [36,66,67].

Glial involvement has also been hypothesized in the transformation of migraines to chronic daily headaches and medication overuse headache (MOH) [64,65]; the use of glial modulators for the management of these headaches has therefore been proposed. However, in a small double-blind, randomized, placebo-controlled trial in patients with chronic migraine, ibudilast, a cAMP phosphodiesterase (PDE) inhibitor and glial cell modulator that has shown to decrease the production of pro-inflammatory cytokines and gliosis [68,69], did not improve migraine symptomatology or decrease frequency [70]. In addition, in another small study, ibudilast did not improve headache or reduce opioid use in patients with MOH [71].

The strongest evidence of glial involvement in headache disorders comes from studies in astrocytes [55,58]. Serum levels of S100B, a calcium protein produced primarily in astrocytes but also in Schwann cells, are increased in children and adults with migraine [72,73,74]. It has been shown in familial hemiplegic migraine 2 (FHM2), a rare subtype of migraine with aura, that mutations in the expression of *Atp1a2* cause ‘loss of function’ of the Na+/K+ ATPase pump in astrocytes, therefore affecting glutamate clearance [75]. Cortical spreading depression (CSD) is believed to be the underlying mechanism of migraine aura [76,77]. FHM2-knockin mice exhibit increased glutamate levels, which facilitate the induction of CSD [78]. FHM2-knockin mice also exhibit increased dendritic excitability in cingulate cortex pyramidal neurons and increased sensitivities to head pain triggers, which can be rescued by re-expression of the *Atp1a2* gene in astrocytes within the cingulate cortex [79]. Tonabersat, a gap-junction modulator that inhibits CSD in animal models [80], is also known to inhibit neuronal-SGC signaling [81]. In clinical trials for migraine prophylaxis, tonabersat has been shown to have some efficacy [82,83,84], particularly in migraine with aura. Neuronal activation may cause transient extracellular alkalosis, which is buffered by glia acid secretion via electrogenic Na+-HCO3−contransporter NBCe1; loss of NBCe1 activity in astrocytes causes dysregulation of synaptic pH [85]. Changes in pH affect neuronal excitability, which has been shown in familial hemiplegic migraine [86]. Furthermore, evidence has suggested that adenosine signaling may play a role in migraine [15]. Astrocyte-derived adenosine is critical for glutamate homeostasis; activation of the adenosine A2A receptor by adenosine regulates glutamate transport via glutamate transporter 1 in astrocytes [63]. In addition to neuron–astrocyte interactions, astrocyte interaction with the vasculature has been shown. Astrocytes are in close contact with vascular cells and have the ability to influence vascular tone and release mediators that could result in either vasoconstriction or vasodilation [87,88]. Lastly, in a comprehensive genome-wide association study (GWAS) that examined the contribution of synaptic genes and glial genes (astrocytes, microglia, and oligodendrocytes) in migraine patients with and without aura, genes found to be associated with migraine are predominantly those involved in signal transduction and protein modification in astrocyte and oligodendrocytes [89].

## 3. TMD

TMD is a musculoskeletal dysfunction within the masticatory system comprising muscle pain and TMJ inflammation [90,91]. TMD pain commonly resides in the masseter muscle, preauricular area and anterior temporalis muscle region [90,91]. This disease, which is characterized by motion disorder, muscle pain, and inflammation of the joint, afflicts approximately 33% of the population and is highly prevalent in individuals between the ages of 20 and 40 years old [90]. Individuals with symptoms of TMD experience catching or locking of the joint, masseter pain, masticatory stiffness, a restricted mandibular range of motion, and TMJ dislocation, all of which considerably impair the quality of life [90]. The preceding examination has revealed that TMD symptoms can arise from numerous factors such as oral deformities, eating hard or chewy foods, yawning wide, clenching teeth, holding tension in the masticatory muscles and stress [90]. Pain symptoms that develop in the TMJ involve local inflammation associated with immune cell activation that induces neuronal sensitization through the release of pro-inflammatory mediators such as TNFα, IL1β, and IL6 [92,93,94]. In addition, it has been highlighted that both peripheral and central glial cells contribute to the physiopathology of TMD.

TMD has been largely studied using rodent models that consist of the injection of pro-inflammatory mediators, such as Complete Freund’s Adjuvant (CFA), carrageenan, or mustard oil in the TMJ [23,35,37,95]. Because CFA injection in the TMJ is the most used model to study TMD, this section will focus on glial cell dysregulation in models of CFA-induced TMJ inflammation.

### 3.1. Glial Cell Activation in the TG in Rodent Models of CFA-induced TMJ Inflammation

CFA injection into the TMJ increases the expression of SGC activation marker glial fibrillary acidic protein (GFAP) in the TG; SGC activation is observed within a few hours after the induction of the inflammation and lasts for at least one week [23,24]. SGC activation is associated with the increase of receptor expression that enhances SGC sensitivity to extracellular signals and parallels pain symptoms. Magni et al. [24] showed an overexpression of P2Y1 and P2Y2, two purinergic receptors important for SCG activation. The stimulation of SGCs with the neuropeptide CGRP potentiated calcium response induced by P2Y2 agonist UTP, contributing to downstream sensory neuron hyperactivity [96]. Moreover, the pharmacological blockade of P2Y2 reduced hyperalgesia in CFA-induced TMJ inflammation [96]. However, the administration of the P2Y1 antagonist failed to reduce hyperalgesia in the same model, suggesting a differential involvement of SGC P2Y1 and P2Y2 receptors in sensory neuron sensitization and hyperalgesia in the temporomandibular area [24].

The inflammation of the TMJ also affects SGC-sensory neuron communication through dysregulation of connexins, the gap junction-forming proteins that allow small molecules such as cAMP, glutamate and ions to directly activate neighboring cells. CFA injection into the TMJ induces overexpression of connexins 36 and 40 in neurons, connexin 43 in SGCs, and connexin 26 in both SGCs and neurons, increasing the permeability between gap junction-connected cells [22,37]. In addition, selective inhibition of connexin 43 was shown to reduce CFA-induced upregulation of GFAP, connexin 43, IL1β, Nav1.7, and mechanical hypersensitivity, indicating that connexin 43 can modify neuronal and SGC activity [22]. The increase of neuronal–glial cell signaling through gap junctions has been shown to increase neuronal excitability in various pain models [37,97,98], supporting the idea that SGCs contribute to sensory neuron sensitization through connexin expression/ function in TMJ inflammation.

Another characteristic of activated SGCs in TMD physiopathology is the production and release of pro-inflammatory mediators [25,26,34]. CFA injection in the TMJ increases COX2 expression and subsequent production of PGE2 by SGCs. PGE2 activates EP2 receptors to increase the expression of the voltage-dependent sodium channel Nav1.7 in TG neurons, leading to neuronal hyperexcitability [25,26,27]. COX2 inhibition or the blockade of SGC activation with fluorocitrate reduces Nav1.7 upregulation and hyperalgesia after CFA injection in the TMJ [25,26,27]. Similar to PGE2, IL1β expressed by SGCs can increase Nav1.7 expression in TG neurons [25].

### 3.2. Microglia and Astrocyte Activation in the TNC in Rodent Models of TMJ Inflammation

The injection of pro-inflammatory mediators into the TMJ induces central sensitization, which is accompanied by microglia and astrocyte activation [29,30,32,35,99]. In contrast to the microglia marker Iba1, which is upregulated as early as 24h after the injection of inflammatory mediators in the TMJ [32,33,35], upregulation of the astrocyte marker GFAP at early time points are not consistent [23,28,30,100]. However, all studies evaluating GFAP expression in the TNC later than 3 days after TMD induction showed an upregulation of GFAP, suggesting that astrocytes are more important in the persistence of CFA-induced TMJ inflammation [28,29,30].

Activated central glial cells contribute to CFA-induced neuronal sensitization through similar mechanisms as SGCs in the TG. CFA injection induces mechanical hyperalgesia, astrocyte activation and overexpression of connexin 43 and IL1β. Intracisternal injections of glial cell inhibitors reduce the expression of connexin 43, IL1β, the neuronal sensitization marker pNR1 in the TNC, and mechanical hyperalgesia induced by CFA [28,30]. CFA injection also activates microglia in the TNC, which subsequently overexpress TNFα, contributing to the development of pain symptoms [34].

### 3.3. Glial Cells Activation in Other Animal Models of TMD

Whereas CFA is typically used for modeling tonic/chronic pain, other pro-inflammatory agents, such as carrageenan, zymosan, capsaicin, and formalin, are used to model acute TMJ pain. Similar to CFA, carrageenan, zymosan, capsaicin, or formalin injections in the TMJ increase the expression of the microglial markers Iba1 and CD11b in the TNC [35,37,38,101]. Capsaicin and formalin injections into the TMJ induce increased p38 phosphorylation in microglia in the TNC and expression of connexin 26, 36, and 40 in SGCs in the TG [37,38,102].

Besides TMJ inflammation models, other TMD models that are more clinically relevant have been developed. In TMD models induced by sustained mouth opening, masseter tendon ligation, experimental tooth movement and stress, both peripheral and central glial cell activation have been shown [39,40,41,42,103]. The expression of astrocyte marker GFAP and microglia marker CD11b increased in both the TG and the TNC, and glial inhibitors reduced hyperalgesia in the temporomandibular area in these non-CFA induced TMD models [40,41]. Chronic stress increased the expression of IL1β in SGCs, and the local administration of the SGC inhibitor L-α-aminoadipate into the TG reduced the overexpression of IL1β and mechanical hyperalgesia [41]. Zhao et al. [42] showed that chronic stress induces persistent astrocyte activation but not microglia activation in the TNC that parallels the development of mechanical hyperalgesia. Moreover, whereas administration of the astrocyte inhibitor L-α-aminoadipate reduced stress-induced hyperalgesia, the microglia inhibitor minocycline was ineffective at reducing pain symptoms [42], suggesting a specific contribution of astrocytes in the central mechanism underlying stress-induced TMD in animal models.

## 4. Dental Pulp Injury

The dental pulp is a unique organ with complex neurobiology in which the afferents innervating pulp play a critical role in modulating immunological, repair, and regenerative functions [104,105]. Although there is much to be discovered about the physiological role of glial cells, specifically within the dental pulp, the importance of glial cells in the maintenance and repair of peripheral afferents is established, especially in the setting of injury. For example, cytokines and neurotrophic factors released from Schwann cells play a critical role in peripheral regeneration after cut or crush injury to peripheral nerves [106]. The dental pulp is densely innervated, and pulpal afferents exhibit a dynamic response upon different types of injury [107]. Although a direct causal effect of pulpal Schwann cells has not yet been demonstrated, elegant studies from Couve and colleagues have shown that their expression is dynamic during the physiological process of pulpal deafferentation that occurs when primary teeth are shed in humans [108]. A recent study demonstrated that pulpal Schwann cells interact with macrophages and are capable of inducing macrophages into the anti-inflammatory M2 phenotype [109]. This raises the intriguing possibility that pulpal Schwann cells play a protective role during inflammatory processes. Dental pulp Schwann cells also function as a reservoir of multipotent mesenchymal stem cells that can differentiate into odontoblasts, which are specialized pulp cells critical to hard tissue repair in teeth [110]. In addition to the dynamic role within the pulp, glial cell expression and activity within the TG and the TNC are responsive to dental pulp inflammation and injury and may play a role in persistent pain after pulp injury [8,9,10,111,112]. Interestingly, glial cell activation following pulp inflammation could lead to increased excitabilities of adjacent uninjured TG neurons, which could explain the clinical phenomena of ectopic tooth pain, in which pain originating from a single inflamed dental pulp, can be difficult to localize and produce pain in other non-injured orofacial structures [8,9,46,112,113]. Collectively, peripheral and central glial cells are important to the basic physiology of the dental pulp and the peripheral and central nervous system responses to injury and inflammation of the pulp.

### 4.1. Glial Cells within the Pulp

The primary glial cell types found within the dental pulp are myelinating and non-myelinating Schwann cells, and they surround the dense array of highly branched afferent fibers innervating pulpal tissue. Within the dental pulp, when evaluating the histological appearance of the afferents in cross-sections, the vast majority of fibers appear to be non-myelinated C-fibers [114]. However, many or most of these afferents are in actuality originating from medium and large diameter myelinated TG neurons [115,116]. It appears that as the afferents ascend through the root, into the crown, and further to the most peripheral aspect of the pulp, the afferents branch extensively, narrow, and lose their myelin [117,118]. There are several other unique aspects of pulp innervation, including the high density of the afferents within the tissue and the observation that despite many of the afferents having the phenotype of putative non-nociceptive Aβ fibers, nearly any activation of pulpal afferent elicits pain [119].

The presence of Schwann cell in the dental pulp has been evaluated using markers S100B and GFAP [108,118]. The non-myelinating Schwann cells are located adjacent to pulpal afferents in the most peripheral aspect of the pulp, where the afferents have branched extensively, and the soft pulp tissues interface with the hard tissue of dentin. The myelinating Schwann cells are located in the pulp tissues of the root, inferior to the more coronal branching location, and co-localize with markers of myelin protein such as myelin basic protein [118]. Interestingly, markers of myelinated afferents, such as neurofilament 200, do not always co-localize with the myelin basic protein in the peripheral pulp [118]. This is explained by the phenomenon described above whereby myelinated afferents branch and taper within the pulp tissue and lose their myelin sheath as they travel through the tooth.

Schwann cells within the pulp are dynamic, and their activation is affected by bacterial infection of the adjacent dentin, aging, and physiologic denervation that proceeds primary tooth exfoliation [120]. Studies evaluating Schwann cell markers in human teeth with and without caries have shown that the network of glia in the pulp below carious dentin is expanded and has altered morphology [120,121]. As caries encroaches into the pulp, the glial network breaks down [121]. This glial network might provide a biological barrier protecting the pulpal tissues from the bacterial pathogens and their by-products, as well as engage in the immune response. For example, Schwann cells may induce macrophages into the pro-healing M2 phenotype in the human dental pulp during the repairing process of carious infections [109]. This most peripheral glial network is also likely providing trophic support to the afferents, which are known to sprout into the area of dentinal injury [107]. Physiologic root resorption is the natural process by which primary teeth are shed in preparation for the eruption of the permanent dentition. The process includes coordinated die-back of the axons innervating the primary tooth pulp in a Wallerian-like manner [108]. As the root resorption progresses, the number of myelinated afferents decreases as Schwann cells proliferate and dedifferentiate in the process of clearing the myelin debris [108].

Finally, with aging, the Schwann cell network at the dentin–pulp interface is reduced overall in numbers and complexity, with Schwann cells in aged pulp showing less density and arborization [122]. The diminished Schwann cell network could explain, in part, the reduced capacity for healing and regeneration of the dental pulp with aging [123]. Indeed, aged Schwann cells have a diminished capacity to repair damaged peripheral afferents relative to young Schwann cells [124]. The role of pulpal Schwann cells in regeneration is especially intriguing, as pulpal glial cells can become multipotent mesenchymal stem cells that can then differentiate into pulpal cells, including odontoblasts, the cell type unique to the pulp and responsible for secreting new dentin, the internal hard tissue of the tooth [110]. It is worth noting that in addition to glial cells dedifferentiating into dental pulp stem cells, mesenchymal stem cells from dental pulp also have a unique capacity to differentiate into Schwann cells. For this reason, there is a significant research interest in the therapeutic potential of dental pulp stem cells in the treatment of peripheral nerve regeneration, which hinges on their capacity to differentiate into Schwann cells [125,126]. In summary, pulpal Schwann cells are uniquely positioned to mediate pulpal repair and regeneration.

### 4.2. The Effect of Pulp Injury on Glial Changes in the TG and TN

It is now well known that activation, proliferation, and inflammatory signaling in microglia and astrocytes within the TN and SGCs within the TG are important mechanisms for the maintenance of pathological orofacial pain, as demonstrated using preclinical pain models [127]. More than 20 years ago, Byers and colleagues found a marked upregulation of GFAP staining in the TG after pulp injury, and the work has been replicated by other groups [45,128]. The increased expression of activated satellite glia cells in the TG, as measured by GFAP expression, can occur adjacent to the neurons innervating the injured or inflamed pulp, as well as neurons innervating adjacent, non-injured teeth [113,129]. The increase in GFAP expression occurs more often in small- and medium-sized TG neurons rather than large-sized neurons [129]. The activated SGCs can release an array of pro-inflammatory cytokines, of which IL-1β is important, which can act back on the neuronal cells, producing further activation and sensitization [113]. The connexin proteins, including connexin 43, appear to be important to this spreading phenomenon resulting in the activation of uninjured TG neurons and to ectopic hyperalgesia [46]. However, the impact of glial plasticity in the TG versus the TN on referred pain remains to be demonstrated.

Indeed, there are significant changes in glial architecture within the TN after pulp injury. After an injury to the dental pulp, an increase in GFAP staining occurs ipsilateral to the injury, in both the nucleus caudalis and the transition zone between the caudalis and interpolaris of the TN, suggesting astrocytic involvement [10]. Increased astrocyte glutamine synthetase activity initiates central sensitization and contributes to nociceptive behavior in rats with both acute and chronic pulp injury [43,44,47]. It is also observed that Iba1 is increased ipsilateral to tooth injury in the transition zone, suggestive of a role for microglia in central changes after dental pulp injury [10]; inhibitors of microglial p38MAPK activities blocks central sensitization in the TN [44]. These findings are consistent with those of Gobel and Brink, who described the involvement of glia in the removal of axonal debris from degraded pulpal afferent terminals, after pulp injury, in the TN [130].

Collectively, the demonstration of increased glial markers in the TN with dental pulp injury provides a plausible mechanism for persistent post-treatment pain after tooth infection and pulp injury. There is now good clinical evidence that persistent pain can occur after routine dental clinical interventions performed to address dental pulp inflammation, including root canal treatment and tooth extraction [111,131]. Although there is more work to be done to understand the biological mechanisms behind persistent dental pain, it is possible that pathological changes of glial cells within the TN or TG could be an important contributory mechanism.

## 5. HNC

HNC is the sixth most common cancer worldwide, causing significant morbidity and mortality [132]. HNC accounts for more than 650,000 cases and 330,000 deaths annually worldwide [132]. Pain is one of the most significant morbidities associated with HNC. Up to 85% of HNC patients report pain at the diagnosis, and most receive inadequate pain treatment [133,134]. Pain in the orofacial region due to cancer impairs an individual’s speech, swallowing, eating, drinking, and interpersonal skills, having a drastic impact on their quality of life [135]. Inflammation and nerve injury caused by nerve compression and tissue destruction contribute to HNC pain [11]. The cancer microenvironment produces mediators that activate and sensitize primary afferent neurons, contributing to the onset and sustainability of cancer pain in the orofacial region [136,137,138,139]. The role glial cells play in mediating the etiology of HNC pain remains poorly understood.

In animal models of HNC-induced pain, data on glial activation are mixed. In a face cancer model produced by Walker 256B breast cancer cells inoculated into the rat vibrissa pad, the upregulation of microglia and astrocyte markers but not SGC markers were found in the ipsilateral TNC, indicating that glial activations occurred only in the CNS [50]. Inhibiting central glial activation by propentofylline suppressed allodynia, hyperalgesia, and spontaneous pain induced by face cancers [50]. Using the same model, the same group of researchers examined the time course of microglia and astrocytes activation, as well as the effect of propentofylline on behavioral outcomes. The study demonstrated transient activation of microglia and astrocytes in the TNC, and propentofylline was more effective when treated early, suggesting central glial activation might contribute to the development but not the maintenance of pain [51]. In a tongue cancer model produced by oral squamous cell carcinoma cells (SCC-158) inoculation, rats developed local mechanical and heat sensitivity, accompanied by hypersensitivity in the spinal wide dynamic range (WDR) neurons, microglia activation, and P2Y12 expression in the TNC [49]. Administration of P2Y12 antagonist or minocycline reversed associated nociceptive behavior, microglial activation, and WDR neuron activity [49]. In contrast, in a rat gingiva SCC model using the same cell line SCC-158, the number of SGCs encircling the medium and large neurons was increased in the TG, accompanied by mechanical allodynia in the whisker-pad skin induced by the cancer growth [48]. However, microglia and astrocytes in the TNC were not activated in the gingiva cancer model [48]. These differences in glial activation patterns may reflect differences in anatomical location, the cell line used, or time course in tumor development and glial activation. Nevertheless, these studies suggest that glial activation could modulate neuronal activities and nociceptive behaviors in mice with HNC.

Perineural invasion, an active process that cancer invades into the nerve, occurs frequently in HNC patients and is associated with increased pain [11]. Emerging evidence highlights a critical role of Schwann cells in perineural invasion. Schwann cells are activated in the presence of pancreatic, colon, gastric, lung, skin, and oral cancer cells and promote cancer growth, invasion, and dispersion [11,52,140,141,142,143,144,145,146,147,148,149]. Cancer-activated Schwann cells secrete neurotrophic factors, chemokines/cytokines, proteases, and adhesion molecules [52,143,146,147,149,150], many of which could directly excite and sensitize primary afferent neurons [151,152,153]. We and others recently found that Schwann cells and oral cancer cells reciprocally interact to promote each other’s growth, migration, and invasion [52,147,152]. Besides, supernatant collected from oral cancer-activated Schwann cells produces a nociceptive response in mice [52]. In our in vivo model of perineural invasion, oral SCC cells invading into the sciatic nerve induced nerve injury and Schwann cell abnormalities, which were accompanied by mechanical hypersensitivities and spontaneous pain in mice [11]. These findings highlight the importance of Schwann cells in cancer progression and associated pain.

## 6. Remaining Questions and Future Directions

Glial activation mechanisms: Research in the field has focused on glial activation patterns using immunostaining of glial activation markers and examining glial cell hypertrophy or proliferation following inflammation or tissue injury [6]. The molecular mechanisms of glia activation and the time course of glial activation have not been explicitly studied in orofacial pain conditions. Elucidating such mechanisms might help hijack glial activation programs for better pain control.

Mediators released by glial cells: Understanding the synthesis and release of glial mediators (e.g., cytokines, chemokines, growth factors, and proteases) to the extracellular space under normal and pathophysiological conditions is key to understand glia–glia interaction, neuron–glia interaction, glia–immune cell interaction, and glia–cancer interaction. Chronic orofacial pain is a dynamic process; understanding the changes of glia mediators during the time course of the pathological pain states is also important.

The role of peripheral glia: The role of peripheral glia, especially Schwann cells, in orofacial pain remains poorly understood, particularly in headache and TMD conditions. Activated Schwann cells secrete cytokines, chemokines, and neurotrophic factors that can activate and recruit immune cells, disrupt the blood-nerve barrier, promote axonal growth, and contribute to abnormal pain states during inflammation and nerve injury [154,155,156,157]. Recently, studies have shown that specialized Schwann cells located at the skin terminals of C-nociceptive fibers can be directly activated by noxious stimuli [158]. TRPA1, an ion channel implicated in many orofacial pain conditions including TMD [91,159], is also expressed in Schwann cells, and the selective deletion of TRPA1 in Schwann cells reduces oxidative stress, macrophage infiltration, and sustained mechanical pain in sciatic nerve ligation and alcohol-induced neuropathic conditions [159,160,161].

Sex differences: Pain is more prominent in women with chronic orofacial pain conditions such as headache, TMD, pulpal injury, and HNC [6,162]. Sex differences in microglia in response to nerve injury have been nicely demonstrated in mice. Spinal microglial signaling inhibitors, such as minocycline and p38 inhibitors, reduce neuropathic pain primarily in male mice in a testosterone-dependent mechanism, with minimal effect on female mice [6,163]. In rodents with orofacial pain, the contribution of glial cells in sex dimorphism is still an open question. Whether other glial cells, such as astrocytes, SGCs, and Schwann cells, play a role in sex dimorphism in orofacial pain needs to be investigated.

Glia and pain resolution: Glia activation is not always bad. Activated glia also release anti-inflammatory mediators and neuroprotective factors that are important for nerve regeneration, repair, and pain resolution [164,165]. Understanding the molecular switch between the pro-nociceptive and the anti-nociceptive state of glial activation could be a powerful strategy to treat orofacial pain [164].

Body region/ pain type differences: The contribution of glial cells in pain is not as extensively studied in the orofacial region compared to other body regions [6]. Neurons and glial cells exhibit diverse functional and anatomical changes that are disease-specific [166]. While DRG and TG neurons share some functional and molecular similarities, they differ in embryonic origins, transcription patterns, signaling pathways, and responses to certain drugs [167,168,169]. A better understanding of the similarities and differences in glial mechanisms across different body regions and pain conditions will help develop targeted therapies to improve efficacy and minimize unwanted side effects.

Glial activation in higher brain centers: The majority of the studies so far have focused on glial activation in the TG and TNC in orofacial pain. The pain circuit is not complete without ascending pathways from the TNC to higher brain centers and the descending modulatory pathways from higher brain centers [9,168,170]. Chronic pain is associated with functional, structural plasticity and reorganization of the pain circuits [166]. Glial plasticity has been shown in the thalamus, the descending modulatory pathway as well as the limbic systems in a few orofacial pain conditions [8,31,170,171,172,173,174]. How glial cells modulate neuronal plasticity both functionally and structurally and their role in the organization of pain circuits at different disease states remains a daunting task in the orofacial pain field.

Glia–immune cell interactions: It is well known that the immune system plays a critical role in pain development, maintenance, and resolution [175]. Many types of pain conditions are associated with increased infiltration of immune cells at the site of tissue/nerve injury. The immune cells release autoantibodies, chemokines, and cytokines, influencing neuronal and glial activities at different levels of the pain circuitry [175]. Peripheral immune cells can infiltrate the CNS after injury and may directly alter microglia, astrocytes, and oligodendrocyte function [176]. Microglia are both glia and immune cells; they are resident macrophages of the CNS [127,177,178]. Other glial cells are also known to propagate inflammation, recruit immune cells, and exhibit immune-cell-like properties [127,157,177]. The heterogeneity of immune cell involvement and how they interact with neurons and glia in different orofacial pain conditions remain a considerable challenge for the future.

The glial mechanism in patients: Using Positron Emission Tomography with a radioligand (11)C-PBR28 that binds to the translocator protein (TSPO), a protein unregulated in activated microglia and astrocytes in the brain, studies have probed microglia and astrocyte activation in patients with lower back pain, fibromyalgia, and rheumatoid arthritis [177,178,179,180]. Similar techniques can be applied to patients with chronic orofacial pain to image glial activation in the brain. Peripheral nerve tissue biopsies and postmortem analysis of TG and brain tissues from patients with chronic orofacial pain conditions will also provide insights into glial involvement in chronic orofacial pain. Currently, there are no approved drugs that specifically target glial cells. A few non-selective agents that can either inhibit glia activities or toll-like receptor 4 or glial p38 signaling were explored in clinical trials as potential analgesics. While glial modulators show some promise in certain disease conditions, most clinical studies fail to demonstrate efficacy (Table 2). To improve the efficacy, future works need to resolve the selectivity issues of purported glial modulators. A better understanding of the type of glial cells involved, the timing, and sex dependence of glial contributions to persistent pain in human pain conditions are needed [165].

## Figures and Tables

**Table 1:** Animal models and model-specific glial responses.

Pain Conditions	Animal Models	Glial Responses			
		Schwann Cells	SGCs	Astrocytes	Microglia
**Headache**	Acute/Chronic dural inflammatory soup	ND	− ⇧ pERK [13]	− ⇧ GFAP [14]	− ⇧ OX42 [14] − Release of inflammatory mediators [15,16]
	Cortical Spreading Depression (CSD)	ND		− Maybe involved in initiation and propagation of CSD wave, release of neurotransmitters [17,18,19].	
	Nitroglycerin	ND	− ⇧ pERK, S100 [20]		− Release of inflammatory mediators [21]
**TMD**	CFA injection in the TMJ	ND	− ⇧ GFAP [22,23,24] − Release pro-inflammatory mediators [22,25,26] − ⇧ Gap junction function [22] − ⇧ neuronal activity (Nav1.7 upregulation) [25,26,27]	− ⇧ GFAP [28,29,30,31] − Release pro-inflammatory mediators [28] − ⇧ Gap junction function [28] − Increase neuronal activity (Nav1.7 and pNR1 upregulation) [28,30]	− ⇧ Iba1/CD11b [32,33] − Release pro-inflammatory mediators [34]
	Carrageenan injection in the TMJ	ND	ND	ND	− ⇧ Iba1 [35]
	Zymosan injection in the TMJ	ND	ND	ND	− ⇧ Iba1 [35]
	Capsaicin injection in the TMJ	ND	− S100B [36] − ⇧ Gap junction function [36,37]	ND	ND
	Formalin injection into the TMJ	ND	ND	ND	− ⇧ CD11b [38]
	Masseter tendon ligation	ND	ND	− ⇧ GFAP [39]	− ⇧ CD11b [39]
	Tooth movement	ND	ND	− ⇧ GFAP [40]	− ⇧ CD11b [40]
	Chronic stress	ND	− ⇧ GFAP [41] − Release pro-inflammatory mediators [41]	− ⇧ GFAP [42] − Release pro-inflammatory mediators [42] − ⇧ neuronal activity (pNR1 upregulation) [42]	− Not activated [42]
**Dental pulp injury**	Acute pulp exposure followed by mustard oil application	ND	ND	− ⇧ Glutamine synthase activity [43] − Inhibition of glutamine supply reduces central sensitization [43,44]	− p38MAPK inhibitors reduce central sensitization [44]
	Pulp exposure followed by CFA application	ND	− ⇧ GFAP [45,46] − ⇧ Connexin [45,46]	− Not activated [45]	ND
	Pulp exposure alone	ND	ND	− ⇧ GFAP [10,47] − ⇧ Glutamine synthase activity [47]	− ⇧ Iba1 [10]
**HNC**	Oral cancer cells inoculated into the rat gingiva	ND	− ⇧ GFAP [48]	Not activated [48]	− Not activated [48]
	Oral cancer cells inoculated into the rat tongue	ND	ND	ND	− Activated [49]
	Breast cancer cells inoculated into the rat vibrissa pad	ND	− Not activated [50]	− ⇧ GFAP [50,51]	− ⇧ Iba1 [50,51]
	Oral cancer cells inoculated into themouse sciatic nerve to mimic PNI	− Myelin abnormalities [11]	ND	ND	ND
	Schwann cell supernatant injection	− ⇧ Migration, proliferation, cell size in the presence of oral cancer cells [52]	ND	ND	ND

ND: No Data. Arrows: upregulation.

**Table 2:** Clinical trials using glial modulators.

Drug Name	Target	Indication	Efficacy	Reference
Minocycline	Microglia inhibitor	Third molar surgery	Yes	Gelesko et al., 2011 [181]
		Lumbar discectomy	No	Martinez et al., 2013 [182]
		Diabetic peripheral neuropathy	No	Syngle et al., 2014 [183]
		Carpal tunnel and trigger finger release	No; longer pain in a patient subgroup	Curtin et al., 2017 [184]
		Lumbar radiculopathy	Yes	Vanelderen et al., 2015 [185]
		Unilateral sciatica	No	Sumracki et al., 2012 [186]
Propentofylline	Microglia and astrocytes modulator	Post-herpetic neuralgia	No	Landry et al., 2012 [187]
Ibudilast	cAMP phosphodiesterase inhibitor	Chronic migraine	No	Kwok et al., 2016 [70]
		Medication overuse headache	No	Loggia et al., 2015 [71]
Tonabersat	Gap-junction modulator	Migraine prophylaxis	Yes, in migraine patients with aura	Hauge et al., 2009 [84]
Naltrexone	Toll-like receptor 4 antagonist	Fibromyalgia	Yes	Younger et al., 2009, 2013 [188,189]
Amitriptyline	P38 mitogen-activated protein kinase inhibitor	Lumbar radiculopathy	Yes	Vanelderen et al., 2015 [185]
Losmapimod	P38 mitogen-activated protein kinase inhibitor	Traumatic peripheral nerve injury	No	Ostenfeld et al., 2013 [190]
		Lumbosacral radiculopathies	No	Ostenfeld et al., 2015 [191]
Dilmapimod	P38 mitogen-activated protein kinase inhibitor	Mixed neuropathic pain	Yes	Anand et al., 2011 [192]

## Data Availability

No new data were created or analyzed in this study. Data sharing is not applicable to this article.
